# Context-Dependent Developmental Effects of Parental Shade Versus Sun Are Mediated by DNA Methylation

**DOI:** 10.3389/fpls.2018.01251

**Published:** 2018-08-27

**Authors:** Brennan H. Baker, Lars J. Berg, Sonia E. Sultan

**Affiliations:** Biology Department, Wesleyan University, Middletown, CT, United States

**Keywords:** ecological epigenetics, DNA methylation, non-genetic inheritance, maternal effects, phenotypic plasticity, transgenerational plasticity, shade tolerance, seed provisioning

## Abstract

Parental environment influences progeny development in numerous plant and animal systems. Such inherited environmental effects may alter offspring phenotypes in a consistent way, for instance when resource-deprived parents produce low quality offspring due to reduced maternal provisioning. However, because development of individual organisms is guided by both inherited and immediate environmental cues, parental conditions may have different effects depending on progeny environment. Such context-dependent transgenerational plasticity suggests a mechanism of environmental inheritance that can precisely interact with immediate response pathways, such as epigenetic modification. We show that parental light environment (shade versus sun) resulted in context-dependent effects on seedling development in a common annual plant, and that these effects were mediated by DNA methylation. We grew replicate parents of five highly inbred *Polygonum persicaria* genotypes in glasshouse shade versus sun and, in a fully factorial design, measured ecologically important traits of their isogenic seedling offspring in both environments. Compared to the offspring of sun-grown parents, the offspring of shade-grown parents produced leaves with greater mean and specific leaf area, and had higher total leaf area and biomass. These shade-adaptive effects of parental shade were pronounced and highly significant for seedlings growing in shade, but slight and generally non-significant for seedlings growing in sun. Based on both regression and covariate analysis, inherited effects of parental shade were not mediated by changes to seed provisioning. To test for a role of DNA methylation, we exposed replicate offspring of isogenic shaded and fully insolated parents to either the demethylating agent zebularine or to control conditions during germination, then raised them in simulated growth chamber shade. Partial demethylation of progeny DNA had no phenotypic effect on offspring of shaded parents, but caused offspring of sun-grown parents to develop as if their parents had been shaded, with larger leaves and greater total canopy area and biomass. These results contribute to the increasing body of evidence that DNA methylation can mediate transgenerational environmental effects, and show that such effects may contribute to nuanced developmental interactions between parental and immediate environments.

## Introduction

A fundamental question in understanding phenotypic variation is how organisms integrate environmental cues with inherited biological information to guide development. This information includes more than genes alone, because organisms also inherit environmentally induced developmental factors from their parents, such as altered provisioning of resources to the embryo and epigenetic modifications of genetic material (reviewed by Roach and Wulff, 1987; Herman and Sultan, 2011; Bonduriansky et al., 2012; English et al., 2015). A great deal remains to be determined about both the nature of these inherited developmental effects and their transmission mechanisms.

Initial studies showed that, depending on the plant species, environmentally stressed maternal individuals may either increase or decrease the quantity of nutritive tissues allocated to developing seeds (Haig and Westoby, 1988; Schmitt et al., 1992; Sultan, 1996; Donohue and Schmitt, 1998; Fenner and Thompson, 2005). Such alterations in the amount of provisioning are expected to result in consistently expressed effects on development. For instance, increased provisioning may cause a “silver spoon” effect, endowing progeny with overall growth benefits regardless of their environmental conditions (Grafen, 1988; Uller et al., 2013). In other cases, however, the effects of parental environment on offspring phenotype differ depending on the conditions that offspring themselves encounter (e.g., Miller et al., 2012; Salinas and Munch, 2012). Such context-dependent effects suggest a more targeted form of inherited information, such as epigenetic modifications to specific DNA sites or cytoplasmically transmitted signaling molecules, that can alter gene expression pathways (Jablonka and Raz, 2009; Danchin et al., 2011; Feil and Fraga, 2012; e.g., Scoville et al., 2011) and hence modulate the phenotypic responses of progeny to their own environments (Gapp et al., 2014).

As noted, studies of parental environmental effects on progeny phenotypes have focused largely on the amount of maternal provisioning, which can be easily estimated in most plants by weighing individual seed units or early germinants (Wulff and Bazzaz, 1992; Sultan, 1996; Zas et al., 2013). While changes to cytoplasmic factors are more difficult to test, methods for studying certain epigenetic modifications – in particular DNA methylation – are now well established (Bossdorf et al., 2008; Verhoeven et al., 2016; Richards et al., 2017). In both plants and animals, the addition or removal of methyl groups from cytosine nucleotides at specific loci may be induced by environmental conditions and the altered DNA subsequently transmitted to offspring (e.g., Verhoeven et al., 2010; Dowen et al., 2012; Pastor et al., 2013; Yu et al., 2013; Zheng et al., 2013; Skinner, 2014). Because such DNA methylation state changes can alter patterns of gene activity (reviewed by Law and Jacobsen, 2010; He et al., 2011; Jones, 2012; Schubeler, 2015), they may result in substantial phenotypic consequences (e.g., Zhang et al., 2013; Cortijo et al., 2014; Akkerman et al., 2016; Herman and Sultan, 2016). The role of DNA methylation in mediating inherited environmental effects can be tested by using chemical methyltransferase inhibitors such as 5-azacytidine (Jones, 1985) or zebularine (Cheng et al., 2003) to experimentally reduce methylation (e.g., Bossdorf et al., 2010; Boyko et al., 2010; Herrera et al., 2012; Verhoeven and van Gurp, 2012; Alvarado et al., 2015; Akkerman et al., 2016; Herman and Sultan, 2016). Zebularine causes transient, genome-wide demethylation at levels that can be dosage-regulated (Baubec et al., 2009). It is thus preferable to 5-azacytidine, which has broadly toxic effects and can be biased to specific loci (Cheng et al., 2003; Ghoshal and Bai, 2007; Hagemann et al., 2011).

We investigated inherited developmental effects of shade, a key environmental variable. Because understory shade versus sun is an ecologically critical aspect of plant habitats (Valladares et al., 2016 and references therein), developmental responses of individuals to these alternative environments are an exceptionally well-studied aspect of plasticity both within and across generations (Schlichting and Smith, 2002; Schmitt et al., 2003; Valladares and Niinemets, 2008; Sultan, 2010; Fitter and Hay, 2012; Marin et al., 2018). Plant plasticity to understory shade is distinct from the well-studied adaptive “shade avoidance” syndrome, which is a suite of phenotypic adjustments in response to neighbor shade characterized by reduced branching, slower leaf development, and greater stem and petiole elongation (Dudley and Schmitt, 1996; Smith and Whitelam, 1997). Unlike the shade cast by a neighbor’s shoot, understory shade cannot be easily evaded via plastic avoidance responses such as extending petioles to reposition leaves. Instead, plants generally respond to understory shade by altering phenotypes in ways that maximize light interception under reduced photon flux density, for instance by allocating proportionally more biomass to leaf tissue and producing broader, thinner leaves (Sultan and Bazzaz, 1993; Evans and Poorter, 2001; Navas and Garnier, 2002; Niinemets et al., 2003; Herr-Turoff and Zedler, 2007; Valladares and Niinemets, 2008; Matesanz et al., 2012; Marin et al., 2018).

In addition to these immediate phenotypic adjustments, individual plants may also respond to shaded versus open conditions by modifying their offspring in ways that affect seedling development (e.g., Schmitt et al., 1992; Sultan, 1996; Galloway and Etterson, 2007). As with most cases of inherited environmental effects or *transgenerational plasticity* (Herman and Sultan, 2011; Salinas et al., 2013; Akkerman et al., 2016; Bell and Stein, 2017), the transmission mechanisms for effects of parental shade versus sun remain unclear (McIntyre and Strauss, 2014). Shade habitats are often characterized by specialist taxa with constitutively large seeds, which provide their seedlings with sufficient initial energy reserves to quickly produce a large shoot that affords tolerance of understory conditions (Leishman and Westoby, 1994; Fenner and Thompson, 2005; Leck et al., 2008; Muller-Landau, 2010). If transgenerational effects of shade were based on a similar provisioning mechanism, then, in taxa that inhabit diverse light conditions, shaded parent individuals would be predicted to plastically increase the amount of seed nutritive tissue. Such provisioning effects would likely be consistently expressed, enhancing growth of seedling offspring regardless of their environmental conditions (Haig and Westoby, 1988). However, in several studies, the effects of parental light environment on such functional progeny traits as leaf size and specific area were found to be expressed differently depending on offspring conditions (Galloway and Etterson, 2009; McIntyre and Strauss, 2014), pointing to inherited developmental modifications that more precisely alter progeny development. To date, however, tests have not been conducted in any plant system to determine whether DNA methylation or other epigenetic modifications play a role in mediating the inherited effects of parental shade versus sun.

Here we present the results of a glasshouse experiment testing for inherited effects of parental shade versus sun on progeny developing in alternative (sun and shade) conditions, together with experimental data on the roles of provisioning and DNA methylation in mediating these effects. Our experimental material consisted of naturally evolved (field-based) genotypes of the well-studied plasticity model system *Polygonum persicaria*, a colonizing annual of diverse temperate habitats. Because this species occurs in open, moderately shaded, and patchy light environments (Sultan et al., 1998), variation in parental light conditions may represent an important source of phenotypic variation among and within natural populations. We addressed the following questions: (i) How does parental shade versus sun influence offspring development with respect to ecologically important leaf traits and total seedling growth? (ii) Does parental light environment differently affect seedling development occurring in shade versus in sun? and (iii) Do seed provisioning and/or DNA methylation play a role in mediating inherited effects of shade versus sun on progeny phenotypes?

## Materials and Methods

### Study System

*Polygonum persicaria* is a common herbaceous species introduced from Eurasia to North America by European settlers (Mitchell and Dean, 1978; Staniforth and Cavers, 1979). Experimental genotypes were sampled from three ecologically distinct introduced-range populations: an open, moist pasture (full sun; MHF population, Northfield, MA, United States), a shaded horse paddock (moderate canopy shade; TP population, Dover, MA, United States), and an organic farm (full sun with neighbor shade; NAT population, Natick, MA, United States, site details in Sultan et al., 1998). Field-collected achenes (1-seeded propagules) were inbred under uniform favorable glasshouse conditions for four generations to produce highly inbred (selfed full-sib) genetic lines (hereafter “genotypes”). Because *P. persicaria* has a mixed breeding system with a high degree of natural self-fertilization (Mulligan and Findlay, 1970), such intensively inbred lines can be generated for field-collected genotypes without inbreeding depression (Herman and Sultan, 2016). This allows for a fully factorial design in which replicate plants of each inbred genotype are grown in contrasting parental environments, to produce genetically uniform offspring that differ only in parental environment (Sultan, 1996; Herman et al., 2012; Herman and Sultan, 2016).

### Parental Generation

Fifth-generation inbred achenes of five genotypes (2 MHF, 2 TP, and 1 NAT; see above) were stratified in distilled water at 4°C for 7 weeks, sown into flats of moist vermiculite, and randomly positioned on a glasshouse bench (6/1/12). At the first true leaf stage (4–6 days after emergence; 6/13/12), seedlings of each genotype were individually transplanted into 1 L clay pots filled with a 1:1:1 mix of sterilized topsoil:horticultural sand:fritted clay (Turface^TM^, Profile Products, Buffalo Grove, IL, United States) pre-moistened with 250 mL water. Five days after transplant, two replicate seedlings of each genotype were randomly assigned to one of two parental glasshouse treatments. In the Parental Sun treatment, plants received 100% of incident light (c. 1300 μmol m^-2^ s^-1^ midday photosynthetically active radiation or PAR; Baker, unpublished data), with a Red:Far Red spectral ratio of c. 1.0 (as measured with an SKR R:FR meter; Skye Instruments, Llandrindod Wells, United Kingdom). The Parental Shade treatment consisted of a metal frame covered by 80% neutral-density shade cloth (PAK Unlimited Inc., Cornelia, GA United States) overlaid with strips of green plastic filter (#138, Lee Filters, Burbank, CA United States), providing plants with c. 260 μmol m^-2^s^-1^ midday PAR and a R:FR ratio of c. 0.7, which agrees with measured R: FR ratios under the mixed canopy shade under which annual *Polygonums* occur (Griffith and Sultan, 2005). To simulate natural understory, equidistant holes 3.5 cm in diameter were cut in the shade cloth so that each Parental Shade plant received a daily 15 min sunfleck (Matesanz et al., 2014). Parental plants in both treatments were kept at field capacity moisture and grown for 9 weeks, with bench positions re-randomized weekly. Self-fertilized, full-sib achenes produced by the 10 experimental parents (5 genotypes × 2 parental treatments) were harvested, air dried, and stored at 4°C.

### Offspring Development

Fifty – eighty achenes from each experimental parent were stratified and germinated as described in the section “Parental Generation.” Individual seedlings were transplanted at the first true leaf stage (5/29/15 – 6/1/15) into 200 mL clay pots of 1:1:1 topsoil:sand:fritted clay mix (see section “Parental Generation”). Ten replicate offspring of each experimental parent were randomly assigned to Offspring Sun and Offspring Shade treatments (identical to Parental Sun and Parental Shade treatments; details above), for a total experimental sample of *N* = 200 seedlings (5 genotypes × 2 parental treatments × 2 offspring treatments × 10 replicate seedlings per offspring treatment). Seedlings received 75% sun and were well-watered for 1 day to ensure recovery from transplant shock before they were randomly positioned within treatments and kept at field capacity moisture throughout the experiment.

For each seedling, **stem elongation** (cm from base to apex) was measured after 6, 12, and 19 days in treatment and **leaf number** was counted after 8, 14, and 19 days in treatment. Individual offspring were harvested on day 20 (6/18/15–6/21/15). For each seedling, the two most recent fully expanded leaves were scanned on a LI-3100 leaf area meter (LICOR Inc., Lincoln, NE, United States), oven-dried (at 100°C for 1 h and then at 65°C for ≥48 h), and weighed to estimate specific leaf area (**SLA**: cm^2^ leaf surface area per g leaf tissue) and **mean single-leaf area** (cm^2^). Remaining leaves were separated from stems, and these tissues were oven-dried (at 100°C for 1 h and then at 65°C for ≥48 h) and weighed. **Total leaf area** for each seedling was estimated by multiplying its **SLA** by its total leaf biomass (including the mass of the two leaves sampled for SLA). Root systems were manually washed, dried at 65°C for ≥48 h, and weighed. **Total biomass (g)** was calculated as [total leaf biomass + stem biomass + root biomass], and **% biomass allocation** to each tissue was calculated as [total leaf, stem, or root biomass/total biomass × 100%]. The final sample lacked 14 seedlings due to insufficient germination or abnormal development; in addition 1 seedling was missing data for root mass, 1 seedling was missing data for total leaf area, and 3 outliers were excluded from the analysis (likely due to treatment error): final sample sizes were *N* = 185 for number of true leaves and stem elongation, and *N* = 184 for all other traits.

### Demethylation Experiment

Twenty-four – forty-eight achenes from each experimental parent (genotype × parental treatment combination, see section “Study System”) were individually weighed on a Cahn C-33 microbalance (Cahn Instruments, Cerritos, CA, United States) and stratified in distilled water at 4°C for 5 weeks. The quantity of **seed provisioning** (mg) for each seedling was estimated as initial air-dried achene mass minus air-dried pericarp mass (retrieved after germination).

Chemical demethylation was imposed during germination. Achenes were sown in Petri plates (9/14/16) on solidified 0.8% agar containing either 0 or 45 μM zebularine (hereafter Control and Demethylation germination treatments, respectively). This dose of zebularine had no adverse developmental effects on *P. persicaria* seedlings in a prior study (Herman and Sultan, 2016), and is similar to a dosage used by Baubec et al. (2009) that reduced global 5-methyldeoxycytidine levels by 15–18% in *Medicago truncatula* and *Arabidopsis thaliana*. Petri plates were positioned randomly on a glasshouse bench and re-randomized daily. Each seedling was transplanted 6 days after germinating so that all plants in the Demethylation germination treatment received the same dose of zebularine.

Eight replicate Control and Demethylation seedling offspring of each experimental parent were transplanted (9/23/16–10/4/16) into individual 200 mL clay pots as described in the section “Parental Generation” and placed in a randomized complete block design in an E-7 dual Conviron growth chamber (Controlled Environments Ltd., Winnipeg, MB, Canada) at a 25°C:18°C, 14:10 h day:night cycle. To simulate a uniform understory treatment, the growth chamber was modified with a metal internal frame covered by 30% neutral-density shade cloth (PAK Unlimited Inc., Cornelia, GA United States) lined with green plastic filter (see section “Parental Generation”) with regularly spaced 1 cm circulation holes; experimental seedlings received c. 220 μmol m^-2^ s^-1^ PAR (Baker, unpublished data). Seedlings were kept at field capacity moisture and re-randomized weekly within blocks. The total experimental sample was *N* = 160 seedlings (5 genotypes × 2 parental treatments × 2 germination treatments × 8 replicate offspring per germination treatment).

Seedlings were grown for 25 days before being harvested (10/18/16–10/29/16). At harvest, an overhead photograph was taken of the entire canopy of each seedling and digitized (EasyLeafAreaV2 software; Easlon and Bloom, 2014) to estimate **canopy area**, a functional trait that accounts for leaf overlap. As described in the section “Offspring Development,” a subsample of two leaves was used to estimate **mean single leaf area** before plant tissues were oven dried and weighed to calculate **total biomass**. Eight seedlings were removed from the final sample due to experimental error or abnormal growth, resulting in a final sample size of *N* = 152.

### Data Analysis

All statistical analyses were performed with JMP Pro 13 (SAS Institute, Cary, NC, United States) and graphing was performed with R version 3.3.3 (R Core Team, 2017).

#### Offspring Development

Analysis of Variance (ANOVA) with type III sums of squares was used to analyze the (fixed) effects on each offspring trait of *parental treatment* (PT, Parental Shade vs. Parental Sun), *offspring treatment* (OT, Offspring Shade vs. Offspring Sun), *genotype*, and all two-way and three-way interactions (see Herman et al., 2012 for a similar analysis). *Genotype* was treated as a fixed effect because the genotypes in this study do not represent a random sample of the species’ genetic diversity; rather, the sample was drawn from specific populations in order to encompass the full range of *P. persicaria* light habitats (Sultan et al., 1998, see Herman et al., 2012 for a previous analysis of this genotype sample). To resolve the specific phenotypic effects of parental treatment and genotype within each offspring treatment, separate ANOVAs were performed analyzing the effects of *parental treatment, genotype*, and their interaction on seedling phenotype in each offspring treatment. In the full analysis, **total biomass** was Box-Cox transformed to meet ANOVA assumptions, but transformation was not required for the **total biomass** ANOVA within each offspring treatment, or for any other trait. For each trait, the mean percent change due to Parental Shade compared with Parental Sun (pooled across genotypes) was calculated in each offspring treatment using the equation: 100% × (trait mean**_parentalshade_**–trait mean**_parentalsun_**)/trait mean**_parentalsun_**.

MANOVA was used to test the effects of *parental treatment* (Parental Shade vs. Parental Sun), *offspring treatment* (Offspring Shade vs. Offspring Sun), *genotype*, and all two-way and three-way interactions on **% biomass allocation** to roots, leaves, and stems. To investigate the significant PT × OT interaction effects, separate ANOVA were performed in each offspring treatment analyzing the effects of *parental treatment, genotype*, and their interaction on **% stem**, **% leaf**, and **% root allocation**. Multivariate repeated-measures ANOVA (Scheiner and Gurevitch, 2001) was used to analyze main and interaction effects of PT, OT, and genotype on **stem elongation** and **leaf number** over time. Following a significant sphericity chi-square test, multivariate Wilks’ Lambda was used to assess significance (Cole and Grizzle, 1966).

#### Demethylation Experiment

ANOVA was used to analyze the (fixed) effects on seedling traits of *parental treatment* (PT, Parental Shade vs. Parental Sun), *germination treatment* (GT, Control vs. Demethylation), *genotype*, all two-way and three-way interactions, and spatial *block*. To resolve the distinct effects of Demethylation on offspring of shade and of sun parents, separate ANOVA were performed testing the effects of *germination treatment, genotype*, and their interaction on seedling phenotype in each parental treatment group. For each trait, the mean percent change (pooled across genotypes) due to Demethylation vs. Control germination treatments was calculated in each parental treatment group using the equation: 100 × (trait mean**_Demethylation_**–trait mean**_Control_**)/trait mean**_Control_**. Student’s *t*-test was used to test the effect of *parental treatment* on **seed** **provisioning**. For the full model, **seed provisioning** was also tested as a covariate for **total biomass** but was excluded from the final ANOVA due to non-significance (*p*= 0.1673). The effect of **seed** **provisioning** on **total biomass** was also tested by regression, both for the full sample and within each Parental Treatment × Germination Treatment group.

## Results

### Parental Shade Had Strong, Treatment-Specific Effects on Offspring Traits

**FIGURE 1:**
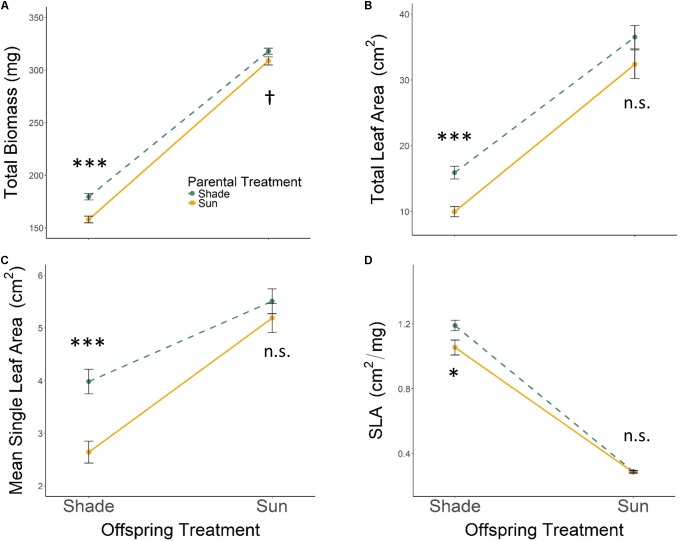
Means ± SE shown (*N* = 184) for **(A)** total biomass, **(B)** total leaf area, **(C)** mean single leaf area, and **(D)** SLA for offspring of Parental Shade (dashed green line) and Parental Sun (solid yellow line) plants that were grown either in Offspring Shade or Offspring Sun treatments. Results of significant tests for the effect of parental shade versus sun in each offspring treatment are shown, based on separate within-treatment ANOVAs (^†^*p* < 0.10, ^∗^*p* < 0.05, ^∗∗∗^*p* < 0.001, n.s. *p* > 0.1, see section “Materials and Methods” for details).

All seedlings grown in Offspring Shade had higher SLA, but lower total biomass, total leaf area, and mean single-leaf area compared to seedlings grown in Offspring Sun (**Figure 1** and **Table 1**, *offspring treatment* *p* < 0.0001 for all four traits). On average, seedling offspring of Parental Shade plants had greater mean values for these four growth traits than offspring of Parental Sun plants (**Figure 1** and **Table 1**, effect of *parental treatment*, *p* ≤ 0.0152 for all traits). For all four traits, these average effects of Parental Shade versus Parental Sun were greater in magnitude than those of genotype (cf. *F*-values, **Table 1**). However, for total biomass, mean single-leaf area, and SLA, the effect of Parental Shade varied significantly depending on the offspring growth treatment (**Table 1**, *PT × OT* interaction effects *p* ≤ 0.0452). In the Offspring Sun treatment, Parental Shade resulted in small, non-significant increases in all four traits compared to Parental Sun (**Figures 1A–D**). For seedlings growing in shade, effects of Parental Shade compared with Parental Sun were dramatic: in the Offspring Shade treatment, progeny of Shade parents produced 44% more total biomass, 60% greater total leaf area, 51% greater mean single-leaf area, and 13% higher SLA than progeny of Sun parents (**Figure 1**; effect of *parental treatment* from ANOVA within Offspring Shade treatment *p*≤ 0.0001, 0.0001, 0.0001, and 0.0188, respectively). These parent-environment effects on total biomass, mean single-leaf area, and total leaf area were greater than the largest *genotype* effects (across treatments) for these traits (*parental treatment* and *genotype* effect *F*-values within Offspring Shade, respectively = 29.9 vs. 5.9 for total biomass; 21.0 vs. 5.8 for single leaf area; 5.7 vs. 1.9 for SLA; and 25.8 vs. 5.6 for total leaf area). For instance, the largest *genotype* effect on total leaf area was a 36% difference between MHF1 and TP2, compared to the 60% greater total leaf area conferred by Parental Shade on average, across genotypes. The *genotype* ×*parental treatment* interaction effect was significant for total leaf area and mean single-leaf area (**Table 1**, *p* = 0.0292 and 0.0536, respectively), and the *genotype* ×*offspring treatment* interaction effect was significant or marginally significant for all traits (**Table 1**, 0.0283 ≤*p* ≤ 0.0879). The three-way interaction (OT × PT × Gen) was non-significant for all four growth traits.

**Table 1:** Results of significance tests for effects of shade versus sun *parental treatment* (PT), shade versus sun *offspring treatment* (OT), and *genotype* (Gen) on seedling traits based on a three-way ANOVA (*N* = 184; details in Materials and Methods).

Source of variation	Total biomass (mg) *R*^2^_adj_ = 0.92		Total leaf area (cm^2^) *R*^2^_adj_ = 0.58		Mean single leaf area (cm^2^) *R*^2^_adj_ = 0.42		SLA (g/cm^2^) *R*^2^_adj_ = 0.83	
	*F*	*p*-value	*F*	*p*-value	*F*	*p*-value	*F*	*p*-value
Parental treatment	24.0282	**<0.0001^∗∗∗^**	11.1616	**0.0010^∗∗^**	13.3534	**0.0003^∗∗∗^**	6.0195	**0.0152^∗^**
Offspring treatment	2177.4669	**<0.0001^∗∗∗^**	222.6994	**<0.0001^∗∗∗^**	87.7163	**<0.0001^∗∗∗^**	867.184	**<0.0001^∗∗∗^**
Genotype	3.705	**0.0065^∗∗^**	3.3402	**0.0117^∗^**	6.3299	**<0.0001^∗∗∗^**	1.9913	0.0982^†^
PT × OT	4.0714	**0.0452^∗^**	0.3937	0.5312	4.9554	**0.0274^∗^**	5.4526	**0.0208^∗^**
Gen × PT	1.5589	0.1877	2.7662	**0.0292^∗^**	2.3824	0.0536^†^	0.67	0.6137
Gen × OT	2.5469	**0.0414^∗^**	3.5605	**0.0082^∗∗^**	2.7876	**0.0283^∗^**	2.0638	0.0879^†^


Significant p-values are shown in bold (^†^p <0.10, ^∗^p < 0.05, ^∗∗^p < 0.01, ^∗∗∗^p < 0.001). The three-way interaction (offspring treatment × parental treatment × genotype) was non-significant for all traits but was included in the model.

**FIGURE 2:**
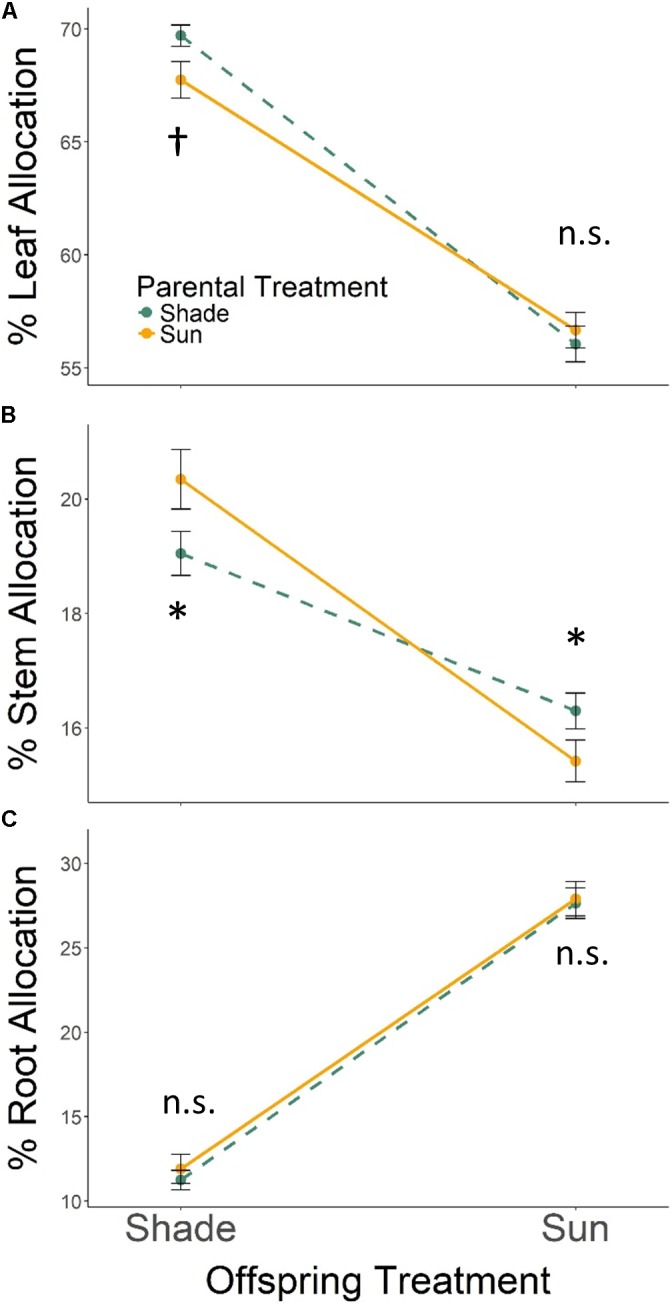
Means ± SE shown (*N* = 184) for **(A)** % leaf, **(B)** % stem, and **(C)** % root biomass allocation for offspring of Parental Shade (dashed green line) and Parental Sun (solid yellow line) plants that were grown either in Offspring Shade or Offspring Sun treatments. Results of significant tests for the effect of parental shade versus sun in each offspring treatment are shown, based on separate within-treatment ANOVAs (^†^*p* < 0.10, ^∗^*p* < 0.05, n.s. *p* > 0.1, see section “Materials and Methods” for details).

With respect to tissue allocation, all seedlings grown in Offspring Shade allocated more biomass to leaf and stem tissues, and less biomass to root tissues, than seedlings in Offspring Sun (**Figure 2**; effect of *offspring treatment* based on MANOVA Wilks’ Lambda *p* < 0.0001). The *parental treatment* effect on biomass allocation varied with *offspring treatment* (PT × OT interaction effect, Wilks’ Lambda *p* ≤ 0.0055): Parental Shade resulted in increased biomass allocation to leaf tissue and lower allocation to stem tissue for progeny growing in Offspring Shade, but did not change leaf allocation, and *increased* stem allocation, for progeny growing in sun (based on ANOVA for each trait within treatments, **Figures 2A,B**). Effects of *parental treatment* on root allocation within each offspring treatment were non-significant (**Figure 2C**). As a result of these progeny treatment-specific effects, the main effect of *parental treatment* on proportional biomass allocation was non-significant (effect of *parental treatment*, Wilks’ Lambda *p* = 0.8647).

**FIGURE 3:**
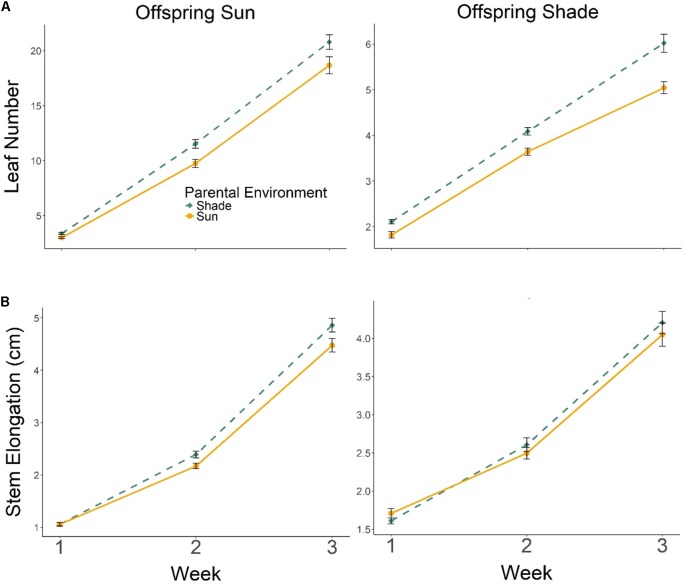
Means ± SE shown (*N* = 185) for **(A)** leaf production and **(B)** stem elongation over 3 weeks growth for offspring of Parental Shade (dashed green line) and Parental Sun (solid yellow line) plants that were grown either in Offspring Shade or Offspring Sun treatments.

Progeny of Parental Shade plants produced more leaves than progeny of Parental Sun plants in both Offspring Sun and Offspring Shade treatments, an effect that increased over time (**Figure 3A**; effect of *parental treatment* ×*time*, multivariate repeated-measures ANOVA Wilks’ Lambda *p* = 0.0136), especially for progeny growing in shade (3-way interaction of *parental treatment* ×*offspring treatment* ×*time*, Wilks’ Lambda *p*= 0.0218; **Figure 3A**). The positive but less pronounced effect of Parental Shade on stem elongation also increased over time in both seedling environments (**Figure 3B**, effect of *parental treatment* × *time* based on multivariate repeated-measures ANOVA, Wilks’ Lambda *p* = 0.0003).

### Partial DNA Demethylation Caused Progeny of Sun Plants to Develop Similarly to Shade Progeny

**FIGURE 4:**
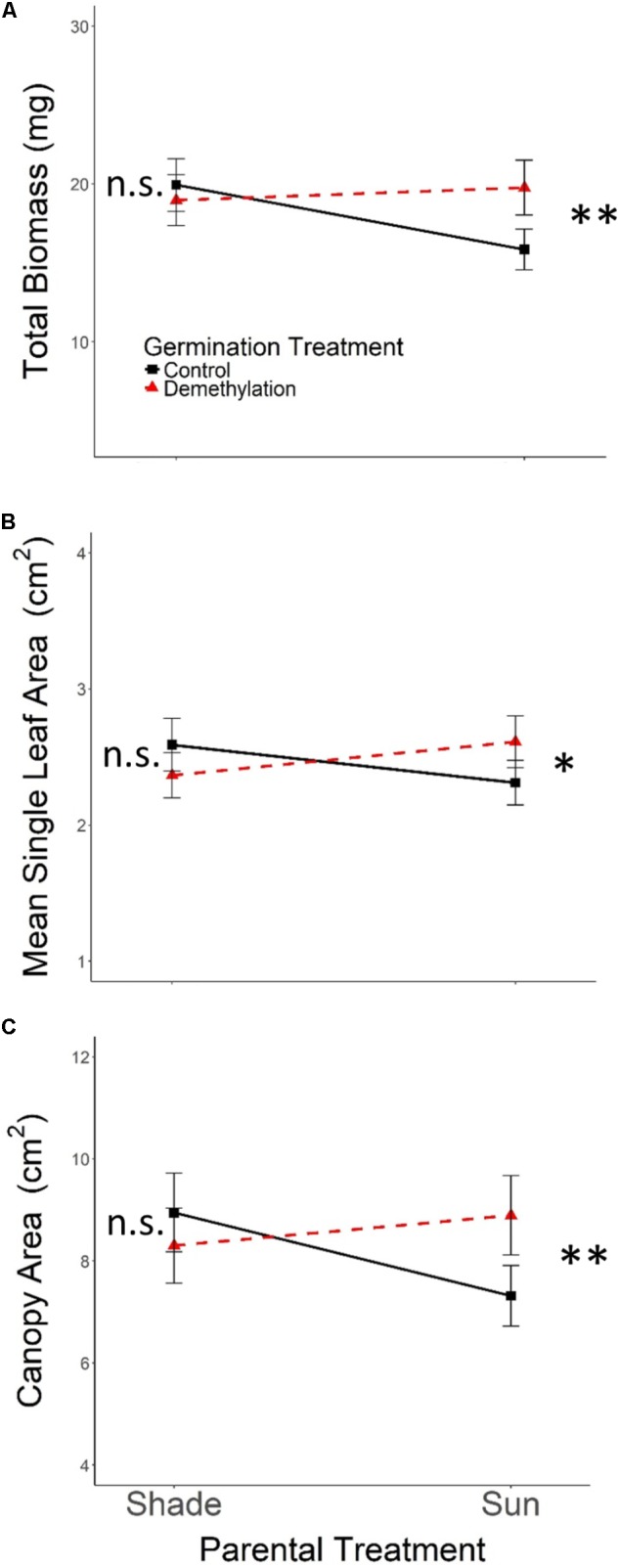
Means ± SE shown (*N* = 152) for **(A)** total biomass, **(B)** mean single leaf area, and **(C)** canopy area for offspring of Parental Shade and Parental Sun plants that were exposed to either 0 μM (Control, solid black lines) or 45 μM zebularine (Demethylation, dashed red lines) during germination. Results of significant tests for the effect of Control versus Demethylation in each parental treatment group are shown, based on separate within-treatment ANOVAs (^∗^*p* < 0.05, ^∗∗^*p* < 0.01, n.s. *p* > 0.1, see section “Materials and Methods” for details).

As expected, the effects of Parental Shade on control-germinated seedlings grown in growth chamber shade in the Demethylation experiment were consistent with parental effects on seedling development in the Offspring Shade glasshouse treatment (described above), where transgenerational effects of parental environment were most strongly expressed: control progeny of Parental Shade plants produced greater total biomass, canopy area, and mean single-leaf area than offspring of Sun parents. The phenotypic impact of Parental Shade versus Sun was substantially altered by partial demethylation with zebularine (**Figures 4A–C**); for all three traits, the demethylation treatment had different effects on Sun and Shade progeny (**Table 2**, *PT* ×*GT* interaction effects; these contrasting effects explain the lack of significant PT and GT main effects). For seedling progeny of Shade parents, demethylation slightly (and non-significantly) reduced biomass, canopy area, and leaf size (5–9% mean trait reductions; **Figures 4A–C**). However, demethylation significantly and substantially altered phenotypic expression in progeny of Sun parents, resulting in seedlings with 25% greater total biomass, 22% increased canopy area, and 13% larger leaves than Control-germinated sun-plant progeny (effect of Control vs. Demethylation *germination treatment* within Parental Sun treatment, *p* = 0.0042 for total biomass, *p* = 0.0448 for canopy area, and *p* = 0.0091 for mean single-leaf area, based on separate ANOVA within each parental treatment). As a result, demethylated progeny of Sun parents (**Figures 4A–C**, red triangles in Parental Sun Treatment) developed very similarly to Control progeny of Shade parents (**Figures 4A–C**, black squares in Parental Shade Treatment). Although genotypes differed significantly on average for all traits (main effect of *genotype*, **Table 2**), 2- and 3-way interaction effects of *genotype* with *parental treatment* and *germination treatment* were non-significant.

**Table 2:** Results of significance tests for effects of shade versus sun *parental treatment* (PT), control versus demethylation *germination treatment* (GT), and *genotype* on seedling traits based on a three-way ANOVA (*N* = 152; details in Materials and Methods).

Source of variation	Total biomass (mg) *R*^2^_adj_ = 0.74		Mean single leaf area (cm^2^) *R*^2^_adj_ = 0.76		Canopy area (cm^2^) *R*^2^_adj_ = 0.76	
	
	*F*	*p*-value	*F*	*p*-value	*F*	*p*-value
Parental treatment	3.1868	0.0767^†^	0.00865	0.9261	1.4398	0.2325
Germination treatment	5.0495	**0.0264^∗^**	1.31143	0.2543	3.2114	0.0756^†^
PT × GT	3.9503	**0.0490^∗^**	3.19571	0.0763^†^	4.8668	**0.0292^∗^**
Genotype	53.439	**<0.0001^∗∗∗^**	83.934	**<0.0001^∗∗∗^**	81.1713	**<0.0001^∗∗∗^**


Significant p-values are shown in bold (^†^p < 0.10, ^∗∗^p < 0.05, ^∗∗∗^p < 0.001). All interactions with genotype were non-significant (but were included in the model).

### Seed Provisioning Did Not Mediate the Growth Effects of Parental Shade vs. Parental Sun

**FIGURE 5:**
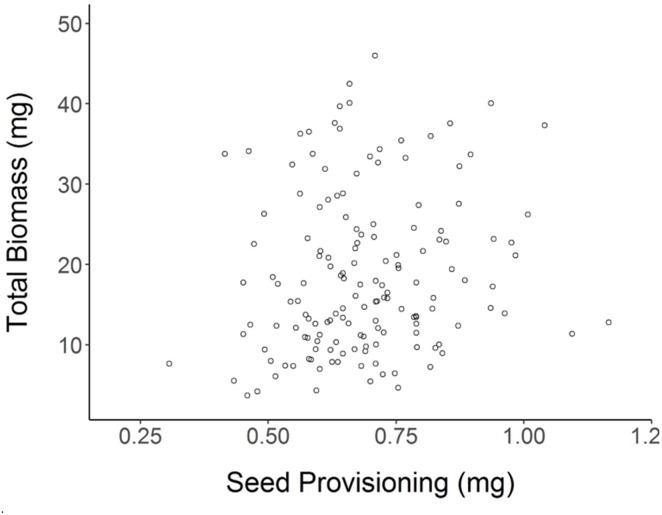
Total biomass of each (day 20) seedling as a function of its initial seed provisioning (*R*^2^_adj_= 0.0154 n.s., *N* = 152; details in section “Materials and Methods”).

Achenes produced by Shade parent plants had 12% lower seed provisioning on average than achenes of Sun parents (Student’s *t*-test for effect of Parental Shade vs. Parental Sun *p* = 0.0002; *N* = 152). Despite this lower seed provisioning, Parental Shade offspring produced greater total biomass and larger leaves than Parental Sun offspring (see previous section). Based on a regression analysis, there was no significant (positive or negative) relationship between seed provisioning and seedling total biomass (**Figure 5**, *R*^2^_adj_= 0.0154, *p* = 0.0687, *N* = 152). Linear regressions calculated separately for each of the 4 *parental treatments × germination treatment* seedling groups were also non-significant (*R*^2^_adj_ ≤ 0.07 in all cases, *p* > 0.05 in all cases) and explained c. 7% of the variation or less within each group.

## Discussion

### Parental Shade Resulted in Specific Alterations to Offspring Phenotypes That Were Functionally Appropriate for Growth in Shade

In isogenic seedlings differing only in parental environment, parental shade versus sun caused specific developmental modifications to offspring: increased allocation to leaf tissues, more rapid shoot development (stem elongation and leaf production), and larger, thinner leaves, resulting in greater total leaf area and seedling biomass. Earlier work on *P. persicaria* also showed specific, but somewhat different, developmental effects of parental shade immediately after germination: after 96 h of growth in a common controlled environment, seedling offspring of shaded parents had produced similar biomass but 30% shorter roots than offspring of full-sun parents, indicating increased proportional allocation to shoot growth during initial development (Sultan, 1996). These data add developmental insights to transgenerational field studies showing that parental light conditions may influence seedling growth and survival in herbaceous species (Galloway and Etterson, 2007; McIntyre and Strauss, 2014).

Increases to light acquisition traits such as leaf biomass allocation, leaf size, and SLA are well known immediate plastic responses to understory shade (Bradshaw, 1965; Bazzaz, 1996; Fitter and Hay, 2012; Sultan, 2015). These allocational, morphological, and structural adjustments are well known to offset the negative growth effects of reduced photon flux density by increasing photosynthetic surface area (Sultan and Bazzaz, 1993; Evans and Poorter, 2001; Navas and Garnier, 2002; Niinemets et al., 2003; Herr-Turoff and Zedler, 2007; Marin et al., 2018; additional references in Valladares and Niinemets, 2008). To our knowledge, the data we report here show for the first time that similar adjustments to these key functional traits can also occur as a result of *inherited* effects of shade experienced by parent plants. In a growing number of plant and animal studies, parent individuals in stressful conditions have been found to produce offspring with specific phenotypic alterations that provide functional adaptation if progeny encounter those same stresses (*adaptive transgenerational plasticity;* e.g., predation, Agrawal et al., 1999, light-limited field microsite, Galloway and Etterson, 2007, drought stress, Sultan et al., 2009, simulated leaf herbivory, Scoville et al., 2011, elevated water temperature, Salinas and Munch, 2012, high dissolved CO_2_ concentration, Miller et al., 2012; additional examples and references in Mousseau and Fox, 1998; Herman and Sultan, 2011; Salinas et al., 2013). A subsequent experimental study of these *P. persicaria* genotypes confirmed that, for progeny that were grown to maturity in either extreme understory or neighbor shade, inherited effects of parental shade were associated with significantly higher lifetime fitness (total reproductive output) compared with parental sun (Baker et al., unpublished).

### Developmental Effects of Parental Shade Versus Sun Varied Depending on Offspring Environment

Although these developmental modifications were qualitatively similar across sun and shade offspring treatments, their degree of expression varied significantly: inherited effects of parental shade versus sun on trait expression were far more pronounced in seedling offspring that were themselves growing in shade. Such context-dependent expression of parental environment effects has been documented in a number of plant and animal taxa (e.g., Schmitt et al., 1992; Galloway, 1995; Hereford and Moriuchi, 2005; Miller et al., 2012; Leverett et al., 2016). In Sheepshead minnow fish (*Cyprinodon variegatus*), for example, the effects of parental temperature treatment were expressed differently depending on the temperature experienced by juvenile offspring (Salinas and Munch, 2012). In plants, such context-specific expression of inherited environmental influences are widespread; the effects of parental drought (Sultan et al., 2009; González et al., 2017), shade (Galloway and Etterson, 2009; McIntyre and Strauss, 2014), nutrient availability (Latzel et al., 2010, 2014), CO_2_ concentration (Lau et al., 2008), salinity (Van Zandt and Mopper, 2004; Vu et al., 2015; Groot et al., 2016; Moriuchi et al., 2016), and temperature (Whittle et al., 2009; Zhang et al., 2012) have all been shown to be expressed differently in alternative offspring environments.

Context-dependent parental effects are captured statistically by significant *parent environment × offspring environment* interaction terms as sources of phenotypic variation. Such complex patterns of expression arise from the various ways that within- and trans-generational environmental influences are integrated by developing organisms (Leimar and McNamara, 2015; Sultan, 2015; Auge et al., 2017). In some cases, favorable immediate conditions in offspring environments may mask or overcome negative transgenerational effects of parental stress. For instance, parental nutrient stress in *Plantago lanceolata* resulted in delayed flowering for progeny in nutrient-poor soil, but this negative developmental effect was not observed when progeny were grown in nutrient-rich soil (Latzel et al., 2014). Conversely, resource-limited progeny environments can mask *positive* parental effects on growth: for instance, parental sun resulted in higher fitness than parental shade when *Claytonia perfoliata* offspring were grown in full-sun, but not when progeny developed in shade, where reproductive output was low regardless of parental light conditions (McIntyre and Strauss, 2014).

In the present case, the more pronounced expression of parental shade effects in offspring that were developing in shade indicates an adaptively integrated response to a particular combination of like inherited factors and immediate cues. Similarly, drought-stressed *P. persicaria* parents produced offspring with an enhanced root extension rate that was further increased when these progeny developed in dry rather than moist soil (Sultan et al., 2009). Investigating the possible selective evolution of this kind of integrated response system is a considerable challenge that researchers are just beginning to approach (Herman et al., 2014; Leimar and McNamara, 2015; McNamara et al., 2016; Sultan, 2016). Such investigations require further information about environmental correlation patterns across generations (Marshall and Uller, 2007; Uller, 2008; Herman et al., 2014), and about other potential sources of variation in the distribution and impact of transgenerational effects, such as differential expression among the progeny of a given parent. For instance, species with complex shoot or inflorescence architectures may evolve position-dependent parental effects on offspring phenotypes. In the closely related annual *P. hydropiper*, which produces achenes at both the axial base and the tip of its flowering spikes, parental shade resulted in shade-adaptive seedling development (faster leaf production and stem extension as well as greater total biomass) in terminal achenes but not in those produced in axillary positions (Lundgren and Sultan, 2005). Such position-dependent expression of parental effects may either provide bet-hedging for uncertain progeny conditions, or allow for alternative phenotypes when progeny are likely to have different dispersal distances from the maternal plant and hence different probabilities of encountering similar microsites (see Donohue and Schmitt, 1998).

### Inherited Developmental Effects of Parental Shade Versus Sun Were Not Mediated by Seed Provisioning

Depending on the species, parent plants in light-limited environments may either increase (Jenner, 1979; Peet and Kramer, 1980) or decrease (Schmitt et al., 1992) the mass of individual seeds, a direct proxy for the amount of endosperm or other nutritive tissues provided to offspring that is often strongly and positively correlated with seedling growth (Roach and Wulff, 1987; Haig and Westoby, 1988, i.e., “silver spoon” *sensu* Grafen, 1988; Uller et al., 2013). In the present study, the progeny of shaded *P. persicaria* parents had slightly lower seed mass on average (after removing the outer pericarp), yet this reduced provisioning was not associated with lower seedling biomass as would be expected in a simple “silver spoon” model for transgenerational effects. Instead, contrary to expectation, the offspring of shaded parents produced *greater* total biomass on average than offspring of full-sun parents, and significantly so for offspring growing in shade. Seed provisioning explained only a very small proportion of variation in seedling biomass, and we found no significant relationship between provisioning and biomass either overall, or within each parent environment-offspring treatment group. Similarly, in an earlier study of *P. persicaria*, isogenic parent plants that were drought-stressed rather than amply watered produced progeny with very different seedling phenotypes, yet seed provisioning (which was similar for both sets of progeny) had no significant effect on variation in either developmental traits or biomass (Herman and Sultan, 2016).

Changes to seed size induced by stressful parental conditions (e.g., Stanton, 1984; Marshall, 1986) have generally been considered the primary mechanism of transgenerational effects on seedling development (Roach and Wulff, 1987; Donohue and Schmitt, 1998; Fenner and Thompson, 2005; while they are not seedling traits *per se*, effects on dormancy and germination have also been intensively studied. However, these result largely from direct changes to maternal [seed coat and fruit] tissues; Penfield and MacGregor, 2017). Results for *Polygonum* suggest that this view be re-examined, since quantity of seed provisions alone may be a less robust predictor of offspring phenotypes than previously believed. To confirm this predictive relationship and infer causation, genetically uniform mother plants must be grown in contrasting conditions and their seeds weighed individually, so that the effect of any resulting seed mass differences on growth traits can be tested using covariate analysis (e.g., Agrawal, 2002; Hereford and Moriuchi, 2005; Herman et al., 2012). When researchers have taken this rigorous approach, results have not always confirmed a major role for provisioning in mediating inherited environmental effects. Using this approach to test transgenerational effects of parental nutrient conditions, for instance, seed provisioning was found to account for most (Stratton, 1989), some (Wulff, 1986; Schmid and Dolt, 1994; Hereford and Moriuchi, 2005; Zas et al., 2013), or none (Wulff and Bazzaz, 1992) of the resulting variation in progeny phenotypes for herbaceous taxa. A second well-studied case is elevated parental CO_2_ concentration, which is well known to result in both increased seed size and progeny growth modifications (Jablonski et al., 2002). A rigorous study by Lau et al. (2008) found that, although maternal CO_2_ concentration strongly affected offspring traits in three different species, there was no evidence that these effects were mediated by seed mass. As in studies of both parental shade versus sun and parental drought versus moist soil in *Polygonum*, the lack of provisioning effects in these cases, despite substantial changes to progeny development, points to an alternative mechanism for mediating inherited effects of parental environment.

The quantity of seed provisioning is only one of several possible factors whereby parental environment may influence progeny phenotypes. Indeed, recent studies of transgenerational effects have revealed a surprisingly diverse set of biological inheritance mechanisms (Day and Bonduriansky, 2011; English et al., 2015). For instance, along with changes to the *quantity* of seed provisioning, parental stresses may induce modifications to the *quality* or composition of seed constituents, including changes in protein content (Parrish and Bazzaz, 1985; Donohue, 2009), hormone concentration (Jha et al., 2010), and stored seed transcripts (Vu et al., 2015). Such changes to inherited signaling molecules may result in specific alterations of progeny development and environmental response pathways, providing a plausible mechanism for adaptively integrated transgenerational effects. Although we found no evidence that changes in seed mass mediate the effects of parental shade versus sun on *Polygonum* offspring, additional studies are needed to determine whether changes to seed constituents involved in regulatory pathways might play a role in this system. Note that changes in the quantity and compositional quality of seed provisioning need not be mutually exclusive; progeny development may be influenced by several types of environmentally induced heritable factors acting cumulatively or interactively (Herman and Sultan, 2011).

### DNA Methylation Changes Play a Role in Mediating the Parental Effects of Shade Versus Sun

Transgenerational effects on progeny may also be mediated by environmentally induced, heritable epigenetic modifications such as changes to methylation state, histone modifications, or non-coding RNAs (Jablonka and Raz, 2009; Sultan, 2015). Because these modifications affect gene activity and hence developmental pathways, they are plausible mediators of context-dependent expression of parental effects. Although other modes of epigenetic transmission may be involved as well (Bonduriansky and Day, 2009; Akkerman et al., 2016), DNA methylation is increasingly viewed as a likely transmission mechanism for transgenerational effects of parental conditions (Kappeler and Meaney, 2010; Herman et al., 2014; Colicchio et al., 2015). In plants, changes in DNA methylation states are known to mediate the effects of several types of environmental stress on progeny phenotypes, e.g., salinity (Boyko et al., 2010), nitrogen deficiency (Kou et al., 2011), drought (Alsdurf et al., 2015; Herman and Sultan, 2016), and herbivory (Akkerman et al., 2016) (additional examples in Bossdorf et al., 2008; Bonduriansky and Day, 2009; Verhoeven et al., 2010, 2016; Herman and Sultan, 2011; Holeski et al., 2012; Richards et al., 2017).

Our experimental demethylation test confirmed that DNA methylation states are involved in mediating transgenerational effects of parental shade versus sun in *Polygonum*. However, the direction of the mediating state change was unexpected. In the few other available studies, chemical demethylation removed the adaptive effects of parental stresses on progeny development, including salt stress in *Arabidopsis thaliana* (Boyko et al., 2010), drought in *P. persicaria* (Herman and Sultan, 2016), and simulated herbivory in *Mimulus guttatus* (Akkerman et al., 2016). In these cases, parental stress apparently leads to stress-adapted progeny via induced *addition* of methyl groups, such that knocking down methylation levels removes the adaptive effect. In this case, by contrast, shade-adaptive progeny phenotypes evidently result from a *removal* of methyl groups that is induced by parental shade: chemically demethylated progeny of sun-grown parents developed the same shade-adaptive features as the progeny of shaded parents, but when progeny of shaded *Polygonum* parents were demethylated, their development was unaltered. To our knowledge, these are the first experimental data showing that adaptive developmental effects of parental stress on progeny can be affected by demethylation rather than addition of methyl groups. These results for parental shade, together with those of Herman and Sultan (2016) for parental drought, show that, even within a given system – here, the same genotypes within a species – adaptive developmental effects of parental stresses on progeny may be established by either methylation or demethylation [i.e., since methylation generally reduces transcriptional activity (Jones, 2012) by either down- or up-regulating relevant components of response pathways].

While these results confirm a role for DNA methylation change in the inheritance of parental shade effects, further molecular work is needed to determine precisely how these effects are transmitted to progeny. Unlike in mammals, where DNA methylation is mostly reset during embryogenesis, methylation states are meiotically stable in plants (Kakutani et al., 1999; Becker et al., 2011; Schmitz et al., 2011). Accordingly, it is possible that shade-induced methylation state changes at loci involved in plastic shade responses may be maintained through meiosis and directly transmitted to offspring. Alternatively, DNA methylation patterns may be reconstructed during embryogenesis (Bouyer et al., 2017) or in developing progeny (Vu et al., 2015) by inherited regulatory molecules (such as hormones, proteins, or non-coding RNAs) that can direct DNA methylation and demethylation (Bonduriansky and Day, 2009; Mahfouz, 2010; Boyko and Kovalchuk, 2011; Zhang and Zhu, 2011; Holeski et al., 2012; Duncan et al., 2014; Matzke et al., 2015). It is also not known whether shade-induced methylation state changes are targeted to specific loci. In this study, genome-wide partial demethylation by zebularine mimicked the parental effects of understory shade on progeny phenotypes, suggesting that parental shade effects may be mediated by similarly non-specific demethylation. Such genome-wide demethylation may result from the loss of methylation marks across cell division (Duncan et al., 2014), for instance due to a shortage of available methyl groups or to reduced activity of DNA methyltransferases (Zhang and Zhu, 2012), perhaps initiated by a metabolic feedback. Although data are not available with respect to shade, other environmental conditions are known to alter these epigenetic regulators (e.g., in *Arabidopsis*, Dowen et al., 2012; reviewed by Meyer, 2015). Alternatively, shade may induce targeted methylation changes, if certain DNA loci are more sensitive than others to changed levels of methyltransferases or signaling molecules. Methylation changes may also interact with changes in the amount or quality of seed provisions (Herman and Sultan, 2011). Assessing the precise roles and relative impact of these inheritance mechanisms is a substantial experimental challenge (Donohue, 2009).

Although it is well established that both biotic and abiotic stresses may induce DNA methylation changes at specific loci (Kovar et al., 1997; Chinnusamy and Zhu, 2009; Dowen et al., 2012) and that these changes may be inherited by descendent generations (Verhoeven et al., 2010; Kou et al., 2011; Zheng et al., 2013), few if any published cases document that these inherited epigenetic changes actually result in tolerance to the inducing stress (Meyer, 2015). Conversely, some studies convincingly link specific epigenetic state changes to adaptive effects, but without demonstrating their stress-induction or heritable transmission (e.g., Xie et al., 2015). Resolving the entire causal pathway, from stress induction, to precise epigenetic changes and their transmission, to phenotypic effects and functional consequences, is a demanding task indeed. More broadly, understanding the mechanisms, dynamics, and adaptive importance of transgenerational effects in plant populations will require not only improved genomic tools for epigenetic studies in non-model species (Richards et al., 2017), but collaborative investigations that draw on molecular, developmental, and ecological expertise.

## Data Availability Statement

The datasets generated and analyzed for this study can be found in the Figshare repository: https://figshare.com/articles/Baker_2018_parental_shade_DNA_methylation_xlsx/6884945.

## Author Contributions

BB, LB, and SS designed the glasshouse experiments, which was conducted by BB and LB. BB and SS designed the demethylation experiments and BB conducted it and carried out the statistical analyses. BB and SS jointly interpreted results and wrote the manuscript.

## Conflictof Interest Statement

The authors declare that the research was conducted in the absence of any commercial or financial relationships that could be construed as a potential conflict of interest.

## References

[B1] Agrawal A. A. (2002). Herbivory and maternal effects: mechanisms and consequences of transgenerational induced plant resistance. *Ecology* 83 3408–3415. 10.1890/0012-9658(2002)083[3408:HAMEMA]2.0.CO;2

[B2] Agrawal A. A. Laforsch C. Tollrian R. (1999). Transgenerational induction of defences in animals and plants. *Nature* 401:60 10.1038/43425

[B3] Akkerman K. C. Sattarin A. Kelly J. K. Scoville A. G. (2016). Transgenerational plasticity is sex-dependent and persistent in yellow monkeyflower (*Mimulus guttatus*). *Environ. Epigenet.* 2:dvw003. 29492285 10.1093/eep/dvw003 PMC5804517

[B4] Alsdurf J. Anderson C. Siemens D. H. (2015). Epigenetics of drought-induced trans-generational plasticity: consequences for range limit development. *AoB Plants* 8:lv146. 10.1093/aobpla/plv146 26685218 PMC4722181

[B5] Alvarado S. Rajakumar R. Abouheif E. Szyf M. (2015). Epigenetic variation in the Egfr gene generates quantitative variation in a complex trait in ants. *Nat. Commun.* 6:513. 10.1038/ncomms7513 25758336

[B6] Auge G. A. Leverett L. D. Edwards B. R. Donohue K. (2017). Adjusting phenotypes via within-and across-generational plasticity. *New Phytol.* 216 343–349. 10.1111/nph.14495 28262950

[B7] Baubec T. Pecinka A. Rozhon W. Mittelsten Scheid O. (2009). Effective, homogeneous and transient interference with cytosine methylation in plant genomic DNA by zebularine. *Plant J.* 57 542–554. 10.1111/j.1365-313X.2008.03699.x 18826433 PMC2667684

[B8] Bazzaz F. A. (1996). *Plants in Changing Environments: Linking Physiological, Population, and Community Ecology.* Cambridge: Cambridge University Press.

[B9] Becker C. Hagmann J. Müller J. Koenig D. Stegle O. Borgwardt K. (2011). Spontaneous epigenetic variation in the *Arabidopsis thaliana* methylome. *Nature* 480:245. 10.1038/nature10555 22057020

[B10] Bell A. M. Stein L. R. (2017). Transgenerational and developmental plasticity at the molecular level: lessons from Daphnia. *Mol. Ecol.* 26 4859–4861. 10.1111/mec.14327 28892281 PMC9437745

[B11] Bonduriansky R. Crean A. J. Day T. (2012). The implications of nongenetic inheritance for evolution in changing environments. *Evol. Appl.* 5 192–201. 10.1111/j.1752-4571.2011.00213.x 25568041 PMC3353344

[B12] Bonduriansky R. Day T. (2009). Nongenetic inheritance and its evolutionary implications. *Ann. Rev. Ecol. Evol. Syst.* 40 103–125. 10.1146/annurev.ecolsys.39.110707.173441

[B13] Bossdorf O. Arcuri D. Richards C. L. Pigliucci M. (2010). Experimental alteration of DNA methylation affects the phenotypic plasticity of ecologically relevant traits in *Arabidopsis thaliana*. *Evolut. Ecol.* 24 541–553. 10.1007/s10682-010-9372-7

[B14] Bossdorf O. Richards C. L. Pigliucci M. (2008). Epigenetics for ecologists. *Ecol. Lett.* 11 106–115. 18021243 10.1111/j.1461-0248.2007.01130.x

[B15] Bouyer D. Kramdi A. Kassam M. Heese M. Schnittger A. Roudier F. (2017). DNA methylation dynamics during early plant life. *Genome Biol.* 18:179. 10.1186/s13059-017-1313-0 28942733 PMC5611644

[B16] Boyko A. Blevins T. Yao Y. L. Golubov A. Bilichak A. Ilnytskyy Y. (2010). Transgenerational adaptation of *Arabidopsis* to stress requires DNA methylation and the function of dicer-like proteins. *PLoS One* 5:e9514. 10.1371/journal.pone.0009514 20209086 PMC2831073

[B17] Boyko A. Kovalchuk I. (2011). Genome instability and epigenetic modification—heritable responses to environmental stress? *Curr. Opin. Plant Biol.* 14 260–266. 10.1371/journal.pone.0009514 21440490

[B18] Bradshaw A. D. (1965). Evolutionary significance of phenotypic plasticity in plants. *Adv. Genet.* 13 115–155. 10.1016/j.pbi.2011.03.003 21440490

[B19] Cheng J. C. Matsen C. B. Gonzales F. A. Ye W. Greer S. Marquez V. E. (2003). Inhibition of DNA methylation and reactivation of silenced genes by zebularine. *J. Natl. Cancer Inst.* 95 399–409. 10.1016/S0065-2660(08)60048-6 12618505

[B20] Chinnusamy V. Zhu J.-K. (2009). Epigenetic regulation of stress responses in plants. *Curr. Opin. Plant Biol.* 12 133–139. 10.1093/jnci/95.5.399 19179104 PMC3139470

[B21] Cole J. Grizzle J. E. (1966). Applications of multivariate analysis of variance to repeated measurements experiments. *Biometrics* 22 810–828. 10.1016/j.pbi.2008.12.006 19179104 PMC3139470

[B22] Colicchio J. M. Miura F. Kelly J. K. Ito T. Hileman L. C. (2015). DNA methylation and gene expression in *Mimulus guttatus*. *BMC Genomics* 16:507. 10.1186/s12864-015-1668-0 26148779 PMC4492170

[B23] Cortijo S. Wardenaar R. Colome-Tatche M. Gilly A. Etcheverry M. Labadie K. (2014). Mapping the epigenetic basis of complex traits. *Science* 343 1145–1148. 10.1186/s12864-015-1668-0 24505129

[B24] Danchin É. Charmantier A. Champagne F. A. Mesoudi A. Pujol B. Blanchet S. (2011). Beyond DNA: integrating inclusive inheritance into an extended theory of evolution. *Nat. Rev. Genet.* 12 475–486 10.1038/nrg3028 21681209

[B25] Day T. Bonduriansky R. (2011). A unified approach to the evolutionary consequences of genetic and nongenetic inheritance. *Am. Nat.* 178 E18–E36. 10.1086/660911 21750377

[B26] Donohue K. (2009). Completing the cycle: maternal effects as the missing link in plant life histories. *Philos. Trans. R. Soc. B Biol. Sci.* 364 1059–1074. 10.1126/science.1248127 19324611 PMC2666684

[B27] Donohue K. Schmitt J. (1998). Maternal environmental effects in plants – Adaptive plasticity? *Mater. Effects Adaptat.* 26 137–158. 10.1098/rstb.2008.0291 19324611 PMC2666684

[B28] Dowen R. H. Pelizzola M. Schmitz R. J. Lister R. Dowen J. M. Nery J. R. (2012). Widespread dynamic DNA methylation in response to biotic stress. *Proc. Natl. Acad. Sci. U.S.A.* 109 E2183–E2191. 10.1073/pnas.1209329109 22733782 PMC3420206

[B29] Dudley S. A. Schmitt J. (1996). Testing the adaptive plasticity hypothesis: density-dependent selection on manipulated stem length in Impatiens capensis. *Am. Natur.* 147 445–465. 10.1086/285860

[B30] Duncan E. J. Gluckman P. D. Dearden P. K. (2014). Epigenetics, plasticity, and evolution: how do we link epigenetic change to phenotype? *J. Exp. Zool. B Mol. Dev. Evol.* 322 208–220. 10.1002/jez.b.22571 24719220

[B31] Easlon H. M. Bloom A. J. (2014). Easy Leaf Area: Automated digital image analysis for rapid and accurate measurement of leaf area. *Appl. Plant Sci.* 2:1400033 10.3732/apps.1400033 25202639 PMC4103476

[B32] English S. Pen I. Shea N. Uller T. (2015). The information value of non-genetic inheritance in plants and animals. *PLoS One* 10:e0116996. 10.1371/journal.pone.0116996 25603120 PMC4300080

[B33] Evans J. Poorter H. (2001). Photosynthetic acclimation of plants to growth irradiance: the relative importance of specific leaf area and nitrogen partitioning in maximizing carbon gain. *Plant Cell Environ.* 24 755–767. 10.1046/j.1365-3040.2001.00724.x

[B34] Feil R. Fraga M. F. (2012). Epigenetics and the environment: emerging patterns and implications. *Nat. Rev. Genet.* 13 97–109. 10.1038/nrg3142 22215131

[B35] Fenner M. Thompson K. (2005). *The Ecology of Seeds.* New York, NY: Cambridge University Press, 1–31. 10.1017/CBO9780511614101

[B36] Fitter A. H. Hay R. K. (2012). *Environmental Physiology of Plants.* Cambridge: Academic press.

[B37] Galloway L. F. (1995). Response to natural environmental heterogeneity: maternal effects and selection on life-history characters and plasticities in mimulus guttatus. *Evolution* 49 1095–1107. 10.1111/j.1558-5646.1995.tb04436.x 28568540

[B38] Galloway L. F. Etterson J. R. (2007). Transgenerational plasticity is adaptive in the wild. *Science* 318 1134–1136. 10.1126/science.1148766 18006745

[B39] Galloway L. F. Etterson J. R. (2009). Plasticity to canopy shade in a monocarpic herb: within- and between- generation effects. *New Phytol.* 182 1003–1012. 10.1111/j.1469-8137.2009.02803.x 19320836

[B40] Gapp K. Jawaid A. Sarkies P. Bohacek J. Pelczar P. Prados J. (2014). Implication of sperm RNAs in transgenerational inheritance of the effects of early trauma in mice. *Nat. Neurosci.* 17:667. 10.1038/nn.3695 24728267 PMC4333222

[B41] Ghoshal K. Bai S. (2007). DNA methyltransferases as targets for cancer therapy. *Drugs Today (Barc)* 43 395–422. 10.1358/dot.2007.43.6.1062666 17612710

[B42] González A. P. R. Dumalasová V. Rosenthal J. Skuhrovec J. Latzel V. (2017). The role of transgenerational effects in adaptation of clonal offspring of white clover (*Trifolium repens*) to drought and herbivory. *Evol. Ecol.* 31 345–361. 10.1007/s10682-016-9844-5

[B43] Grafen A. (1988). “On the uses of data on lifetime reproductive success,” in *Reproductive Success*, ed. Clutton-Brock T. H. (Chicago, IL: University of Chicago Press), 454-471.

[B44] Griffith T. M. Sultan S. E. (2005). Shade tolerance plasticity in response to neutral vs green shade cues in *Polygonum* species of contrasting ecological breadth. *New Phytol.* 166 141–147. 10.1111/j.1469-8137.2004.01277.x 15760358

[B45] Groot M. P. Kooke R. Knoben N. Vergeer P. Keurentjes J. J. Ouborg N. J. (2016). Effects of multi-generational stress exposure and offspring environment on the expression and persistence of transgenerational effects in *Arabidopsis thaliana*. *PLoS One* 11:e0151566. 10.1371/journal.pone.0151566 26982489 PMC4794210

[B46] Hagemann S. Heil O. Lyko F. Brueckner B. (2011). Azacytidine and decitabine induce gene-specific and non-random DNA demethylation in human cancer cell lines. *PLoS One* 6:e17388. 10.1371/journal.pone.0017388 21408221 PMC3049766

[B47] Haig D. Westoby M. (1988). “Inclusive fitness, seed resources, and maternal care,” in *Plant Reproductive Ecology*, eds Doust L. Doust L. (Oxford: Oxford University Press), 60–79.

[B48] He X. J. Chen T. P. Zhu J. K. (2011). Regulation and function of DNA methylation in plants and animals. *Cell Res.* 21 442–465. 10.1038/cr.2011.23 21321601 PMC3152208

[B49] Hereford J. Moriuchi K. (2005). Variation among populations of *Diodia teres* (Rubiaceae) in environmental maternal effects. *J. Evol. Biol.* 18 124–131. 10.1111/j.1420-9101.2004.00797.x 15669968

[B50] Herman J. J. Spencer H. G. Donohue K. Sultan S. E. (2014). How stable ‘should’ epigenetic modifications be? Insights from adaptive plasticity and bet hedging. *Evolution* 68 632–643. 10.1111/evo.12324 24274594

[B51] Herman J. J. Sultan S. E. (2011). Adaptive transgenerational plasticity in plants: case studies, mechanisms, and implications for natural populations. *Front. Plant Sci.* 2:102. 10.3389/fpls.2011.00102 22639624 PMC3355592

[B52] Herman J. J. Sultan S. E. (2016). DNA methylation mediates genetic variation for adaptive transgenerational plasticity. *Proc. R. Soc. B-Biol. Sci.* 283:20160988. 10.1098/rspb.2016.0988 27629032 PMC5031651

[B53] Herman J. J. Sultan S. E. Horgan-Kobelski T. Riggs C. (2012). Adaptive transgenerational plasticity in an annual plant: grandparental and parental drought stress enhance performance of seedlings in dry soil. *Integr. Compar. Biol.* 52 77–88. 10.1093/icb/ics041 22523124

[B54] Herrera C. M. Pozo M. I. Bazaga P. (2012). Jack of all nectars, master of most: DNA methylation and the epigenetic basis of niche width in a flower-living yeast. *Mol. Ecol.* 21 2602–2616. 10.1111/j.1365-294X.2011.05402.x 22171717

[B55] Herr-Turoff A. Zedler J. B. (2007). Does morphological plasticity of the *Phalaris arundinacea* canopy increase invasiveness? *Plant Ecol.* 193 265–277. 10.1007/s11258-007-9264-2

[B56] Holeski L. M. Jander G. Agrawal A. A. (2012). Transgenerational defense induction and epigenetic inheritance in plants. *Trends Ecol. Evol.* 27 618–626. 10.1016/j.tree.2012.07.011 22940222

[B57] Jablonka E. Raz G. (2009). Transgenerational epigenetic inheritance: prevalence, mechanisms, and implications for the study of heredity and evolution. *Q. Rev. Biol.* 84 131–176. 10.1086/598822 19606595

[B58] Jablonski L. M. Wang X. Curtis P. S. (2002). Plant reproduction under elevated CO_2_ conditions: a meta-analysis of reports on 79 crop and wild species. *New Phytol.* 156 9–26. 10.1046/j.1469-8137.2002.00494.x

[B59] Jenner C. (1979). Grain-filling in wheat plants shaded for brief periods after anthesis. *Funct. Plant Biol.* 6 629–641. 10.1071/PP9790629

[B60] Jha P. Norsworthy J. K. Riley M. B. Bridges W. Jr. (2010). Shade and plant location effects on germination and hormone content of Palmer amaranth (*Amaranthus palmeri*) seed. *Weed Sci.* 58 16–21. 10.1614/WS-09-059.1

[B61] Jones P. A. (1985). Altering gene-expression with 5-azacytidine. *Cell* 40 485–486. 10.1016/0092-8674(85)90192-8 2578884

[B62] Jones P. A. (2012). Functions of DNA methylation: islands, start sites, gene bodies and beyond. *Nat. Rev. Genet.* 13 484–492. 10.1038/nrg3230 22641018

[B63] Kakutani T. Munakata K. Richards E. J. Hirochika H. (1999). Meiotically and mitotically stable inheritance of DNA hypomethylation induced by ddm1 mutation of *Arabidopsis thaliana*. *Genetics* 151 831–838. 9927473 10.1093/genetics/151.2.831 PMC1460490

[B64] Kappeler L. Meaney M. J. (2010). Epigenetics and parental effects. *Bioessays* 32 818–827. 10.1002/bies.201000015 20652892

[B65] Kou H. Li Y. Song X. Ou X. Xing S. Ma J. (2011). Heritable alteration in DNA methylation induced by nitrogen-deficiency stress accompanies enhanced tolerance by progenies to the stress in rice (*Oryza sativa* L.). *J. Plant Physiol.* 168 1685–1693. 10.1016/j.jplph.2011.03.017 21665325

[B66] Kovar A. Koukalova B. Bezde M. Opatrn Z. (1997). Hypermethylation of tobacco heterochromatic loci in response to osmotic stress. *Theor. Appl. Genet.* 95 301–306. 10.1007/s001220050563

[B67] Latzel V. Janeček Š Dole žal J. Klimešová J. Bossdorf O. (2014). Adaptive transgenerational plasticity in the perennial *Plantago lanceolata*. *Oikos* 123 41–46. 10.1111/j.1600-0706.2013.00537.x

[B68] Latzel V. Klimešová J. Hájek T. Gómez S. Šmilauer P. (2010). Maternal effects alter progeny’s response to disturbance and nutrients in two *Plantago* species. *Oikos* 119 1700–1710. 10.1111/j.1600-0706.2010.18737.x

[B69] Lau J. A. Peiffer J. Reich P. B. Tiffin P. (2008). Transgenerational effects of global environmental change: long-term CO_2_ and nitrogen treatments influence offspring growth response to elevated CO_2_. *Oecologia* 158:141. 10.1007/s00442-008-1127-6 18716799

[B70] Law J. A. Jacobsen S. E. (2010). Establishing, maintaining and modifying DNA methylation patterns in plants and animals. *Nat. Rev. Genet.* 11 204–220. 10.1038/nrg2719 20142834 PMC3034103

[B71] Leck M. A. Parker V. T. Simpson R. (2008). *Seedling Ecology and Evolution.* Cambridge: Cambridge University Press 10.1017/CBO9780511815133

[B72] Leimar O. McNamara J. M. (2015). The evolution of transgenerational integration of information in heterogeneous environments. *Am. Natur.* 185 E55–E69. 10.1086/679575 25674697

[B73] Leishman M. R. Westoby M. (1994). The role of large seed size in shaded conditions: experimental evidence. *Funct. Ecol.* 8 205–214. 10.2307/2389903

[B74] Leverett L. D. Auge G. A. Bali A. Donohue K. (2016). Contrasting germination responses to vegetative canopies experienced in pre-vs. post-dispersal environments. *Ann. Bot.* 118 1175–1186. 10.1093/aob/mcw166 27551028 PMC5091727

[B75] Lundgren M. R. Sultan S. E. (2005). Seedling expression of cross-generational plasticity depends on reproductive architecture. *Am. J. Bot.* 92 377–381. 10.3732/ajb.92.2.377 21652413

[B76] Mahfouz M. M. (2010). RNA-directed DNA methylation: mechanisms and functions. *Plant Signal. Behav.* 5 806–816. 10.4161/psb.5.7.11695 20421728 PMC3115029

[B77] Marin M. Blandino C. Laverack G. Toorop P. Powell A. (2018). Responses of primula vulgaris to light quality in the maternal and germination environments. *Plant Biol.* [Epub ahead of print]. 10.1111/plb.12849 29788539

[B78] Marshall D. L. (1986). Effect of seed size on seedling success in three species of *Sesbania* (Fabaceae). *American* 73 457–464. 10.1002/j.1537-2197.1986.tb12063.x

[B79] Marshall D. L. Uller T. (2007). When is a maternal effect adaptive? *Oikos* 116 1957–1963. 10.1111/j.2007.0030-1299.16203.x

[B80] Matesanz S. Horgan-Kobelski T. Sultan S. E. (2012). Phenotypic plasticity and population differentiation in an ongoing species invasion. *PLoS One* 7:e44955. 10.1371/journal.pone.0044955 23028702 PMC3446995

[B81] Matesanz S. Horgan-Kobelski T. Sultan S. E. (2014). Contrasting levels of evolutionary potential in populations of the invasive plant *Polygonum cespitosum*. *Biol. Invasions* 16 455–468. 10.1007/s10530-013-0533-9

[B82] Matzke M. A. Kanno T. Matzke A. J. (2015). RNA-directed DNA methylation: the evolution of a complex epigenetic pathway in flowering plants. *Annu. Rev. Plant Biol.* 66 243–267. 10.1146/annurev-arplant-043014-114633 25494460

[B83] McIntyre P. J. Strauss S. Y. (2014). Phenotypic and transgenerational plasticity promote local adaptation to sun and shade environments. *Evol. Ecol.* 28 229–246. 10.1007/s10682-013-9670-y

[B84] McNamara J. M. Dall S. R. Hammerstein P. Leimar O. (2016). Detection vs. selection: Integration of genetic, epigenetic and environmental cues in fluctuating environments. *Ecol. Lett.* 19 1267–1276. 10.1111/ele.12663 27600658

[B85] Meyer P. (2015). Epigenetic variation and environmental change. *J. Exp. Bot.* 66 3541–3548. 10.1093/jxb/eru502 25694547

[B86] Miller G. M. Watson S.-A. Donelson J. M. Mccormick M. I. Munday P. L. (2012). Parental environment mediates impacts of increased carbon dioxide on a coral reef fish. *Nat. Clim. Change* 2:858 10.1038/nclimate1599

[B87] Mitchell R. S. Dean J. K. (1978). *Polygonaceae (Buckwheat Family) of New York State.* New York, NY: University of the State of New York, State Education Department 10.5962/bhl.title.140273

[B88] Moriuchi K. S. Friesen M. L. Cordeiro M. A. Badri M. Vu W. T. Main B. J. (2016). Salinity adaptation and the contribution of parental environmental effects in Medicago truncatula. *PLoS One* 11:e0150350. 10.1371/journal.pone.0150350 26943813 PMC4778912

[B89] Mousseau T. A. Fox C. W. (1998). *Maternal Effects as Adaptations.* New York, NY: Oxford University Press.

[B90] Muller-Landau H. C. (2010). The tolerance–fecundity trade-off and the maintenance of diversity in seed size. *Proc. Natl. Acad. Sci. U.S.A.* 107 4242–4247. 10.1073/pnas.0911637107 20160078 PMC2840174

[B91] Mulligan G. A. Findlay J. N. (1970). Reproductive systems and colonization in canadian weeds. *Can. J. Bot.* 48 859–860. 10.1139/b70-119

[B92] Navas M.-L. Garnier E. (2002). Plasticity of whole plant and leaf traits in Rubia peregrina in response to light, nutrient and water availability. *Acta Oecol.* 23 375–383. 10.1016/S1146-609X(02)01168-2

[B93] Niinemets Ü Valladares F. Ceulemans R. (2003). Leaf-level phenotypic variability and plasticity of invasive *Rhododendron ponticum* and non-invasive Ilex aquifolium co-occurring at two contrasting European sites. *Plant Cell Environ* 26 941–956. 10.1046/j.1365-3040.2003.01027.x 12803621

[B94] Parrish J. Bazzaz F. (1985). Nutrient content of *Abutilon theophrasti* seeds the competitive ability of the resulting plants. *Oecologia* 65 247–251. 10.1007/BF00379224 28310672

[B95] Pastor V. Luna E. Mauch-Mani B. Ton J. Flors V. (2013). Primed plants do not forget. *Environ. Exp. Bot.* 94 46–56. 10.1016/j.envexpbot.2012.02.013

[B96] Peet M. M. Kramer P. J. (1980). Effects of decreasing source/sink ratio in soybeans on photosynthesis, photorespiration, transpiration and yield. *Plant Cell Environ.* 3 201–206.

[B97] Penfield S. MacGregor D. R. (2017). Effects of environmental variation during seed production on seed dormancy and germination. *J. Exp. Bot.* 68 819–825. 27940467 10.1093/jxb/erw436

[B98] Richards C. L. Alonso C. Becker C. Bossdorf O. Bucher E. Colome-Tatche M. (2017). Ecological plant epigenetics: evidence from model and non-model species, and the way forward. *Ecol. Lett.* 20 1576–1590. 10.1111/ele.12858 29027325

[B99] Roach D. A. Wulff R. D. (1987). Maternal effects in plants. *Ann. Rev. Ecol. Syst.* 18 209–235. 10.1146/annurev.es.18.110187.001233

[B100] Salinas S. Brown S. C. Mangel M. Munch S. B. (2013). Non-genetic inheritance and changing environments. *Non-Genetic Inherit.* 1 38–50. 10.2478/ngi-2013-0005 24139597

[B101] Salinas S. Munch S. B. (2012). Thermal legacies: transgenerational effects of temperature on growth in a vertebrate. *Ecol. Lett.* 15 159–163. 10.1111/j.1461-0248.2011.01721.x 22188553

[B102] Scheiner S. M. Gurevitch J. (2001). *Design and Analysis of Ecological Experiments.* New York, NY: Oxford University Press.

[B103] Schlichting C. D. Smith H. (2002). Phenotypic plasticity: linking molecular mechanisms with evolutionary outcomes. *Evol. Ecol.* 16 189–211. 10.1023/A:1019624425971

[B104] Schmid B. Dolt C. (1994). Effects of maternal and paternal environment and genotype on offspring phenotype in *Solidago altissima* L. *Evolution* 48 1525–1549. 10.1111/j.1558-5646.1994.tb02194.x 28568418

[B105] Schmitt J. Niles J. Wulff R. D. (1992). Norms of reaction of seed traits to maternal environments in *Plantago lanceolata*. *Am. Nat.* 139 451–466. 10.1086/285338

[B106] Schmitt J. Stinchcombe J. R. Heschel M. S. Huber H. (2003). The adaptive evolution of plasticity: phytochrome-mediated shade avoidance responses. *Integr. Compar. Biol.* 43 459–469. 10.1093/icb/43.3.459 21680454

[B107] Schmitz R. J. Schultz M. D. Lewsey M. G. O’malley R. C. Urich M. A. Libiger O. (2011). Transgenerational epigenetic instability is a source of novel methylation variants. *Science* 334 369–373. 10.1126/science.1212959 21921155 PMC3210014

[B108] Schubeler D. (2015). Function and information content of DNA methylation. *Nature* 517 321–326. 10.1038/nature14192 25592537

[B109] Scoville A. G. Barnett L. L. Bodbyl-Roels S. Kelly J. K. Hileman L. C. (2011). Differential regulation of a MYB transcription factor is correlated with transgenerational epigenetic inheritance of trichome density in *Mimulus guttatus*. *New Phytol.* 191 251–263. 10.1111/j.1469-8137.2011.03656.x 21352232 PMC3107365

[B110] Skinner M. K. (2014). Endocrine disruptor induction of epigenetic transgenerational inheritance of disease. *Mol. Cell. Endocrinol.* 398 4–12. 10.1016/j.mce.2014.07.019 25088466 PMC4262585

[B111] Smith H. Whitelam G. (1997). The shade avoidance syndrome: multiple responses mediated by multiple phytochromes. *Plant Cell Environ.* 20 840–844. 10.1046/j.1365-3040.1997.d01-104.x 12644683

[B112] Staniforth R. Cavers P. B. (1979). Distribution of habitats of four annual smartweeds in Ontario. *Canad. Field-Nat* 93 378–385.

[B113] Stanton M. L. (1984). Seed variation in wild radish: effect of seed size on components of seedling and adult fitness. *Ecology* 65 1105–1112. 10.2307/1938318

[B114] Stratton D. (1989). Competition prolongs expression of maternal effects in seedlings of Erigeron annuus (Asteraceae). *Am. J. Bot.* 76 1646–1653. 10.1002/j.1537-2197.1989.tb15149.x

[B115] Sultan S. E. (1996). Phenotypic plasticity for offspring traits in *Polygonum persicaria*. *Ecology* 77 1791–1807. 10.2307/2265784 19694132

[B116] Sultan S. E. (2010). Plant developmental responses to the environment: eco-devo insights. *Curr. Opin. Plant Biol.* 13 96–101. 10.1016/j.pbi.2009.09.021 19857987

[B117] Sultan S. E. (2015). *Organism and Environment: Ecological Development, Niche Construction, and Adaptation.* New York, NY: Oxford University Press 10.1093/acprof:oso/9780199587070.001.0001

[B118] Sultan S. E. (2016). “Genotype-environment interaction and the unscripted reaction norm,” in *Cause and Process in Evolution*: *Vienna Series in Theoretical Biology*, eds Uller T. Laland K. (Cambridge, MA: MIT Press).

[B119] Sultan S. E. Barton K. Wilczek A. M. (2009). Contrasting patterns of transgenerational plasticity in ecologically distinct congeners. *Ecology* 90 1831–1839. 10.1890/08-1064.1 19694132

[B120] Sultan S. E. Bazzaz F. (1993). Phenotypic plasticity in *Polygonum persicaria*. I. Diversity and uniformity in genotypic norms of reaction to light. *Evolution* 47 1009–1031. 10.1111/j.1558-5646.1993.tb02132.x 28564281

[B121] Sultan S. E. Wilczek A. Hann S. Brosi B. (1998). Contrasting ecological breadth of co-occurring annual *Polygonum* species. *J. Ecol.* 86 363–383. 10.1046/j.1365-2745.1998.00265.x

[B122] R Core Team (2017). *R: A Language and Environment for Statistical Computing. Vienna: R Foundation for Statistical Computing.* Available at: https://www.R-project.org

[B123] Uller T. (2008). Developmental plasticity and the evolution of parental effects. *Trends Ecol. Evol.* 23 432–438. 10.1016/j.tree.2008.04.005 18586350

[B124] Uller T. Nakagawa S. English S. (2013). Weak evidence for anticipatory parental effects in plants and animals. *J. Evol. Biol.* 26 2161–2170. 10.1111/jeb.12212 23937440

[B125] Valladares F. Laanisto L. Niinemets U. Zavala M. A. (2016). Shedding light on shade: ecological perspectives of understorey plant life. *Plant Ecol. Divers.* 9 237–251. 10.1080/17550874.2016.1210262

[B126] Valladares F. Niinemets Ü. (2008). Shade tolerance, a key plant feature of complex nature and consequences. *Ann. Rev. Ecol. Evol. Syst.* 39 237–257. 10.1146/annurev.ecolsys.39.110707.173506

[B127] Van Zandt P. A. Mopper S. (2004). The effects of maternal salinity and seed environment on germination and growth in Iris hexagona. *Evolut Ecol. Res.* 6 813–832.

[B128] Verhoeven K. J. F. Jansen J. J. Van Dijk P. J. Biere A. (2010). Stress-induced DNA methylation changes and their heritability in asexual dandelions. *New Phytol.* 185 1108–1118. 10.1111/j.1469-8137.2009.03121.x 20003072

[B129] Verhoeven K. J. F. van Gurp T. P. (2012). Transgenerational effects of stress exposure on offspring phenotypes in apomictic dandelion. *PLoS One* 7: e38605. 10.1371/journal.pone.0038605 22723869 PMC3377677

[B130] Verhoeven K. J. F. Vonholdt B. M. Sork V. L. (2016). Epigenetics in ecology and evolution: what we know and what we need to know. *Mol. Ecol.* 25 1631–1638. 10.1111/mec.13617 26994410

[B131] Vu W. T. Chang P. L. Moriuchi K. S. Friesen M. L. (2015). Genetic variation of transgenerational plasticity of offspring germination in response to salinity stress and the seed transcriptome of *Medicago truncatula*. *BMC Evol. Biol.* 15:59. 10.1186/s12862-015-0322-4 25884157 PMC4406021

[B132] Whittle C. Otto S. Johnston M. O. Krochko J. (2009). Adaptive epigenetic memory of ancestral temperature regime in *Arabidopsis thaliana*. *Botany* 87 650–657. 10.1139/B09-030

[B133] Wulff R. D. (1986). Seed size variation in *Desmodium paniculatum*: I. Factors affecting seed size. *J. Ecol.* 74 87–97. 10.2307/2260350

[B134] Wulff R. D. Bazzaz F. A. (1992). Effect of the parental nutrient regime on growth of the progeny in Abutilon theophrasti (Malvaceae). *Am. J. Bot.* 79 1102–1107. 10.1002/j.1537-2197.1992.tb13704.x 30139139

[B135] Xie H. Li H. Liu D. Dai W. He J. Lin S. (2015). ICE1 demethylation drives the range expansion of a plant invader through cold tolerance divergence. *Mol. Ecol.* 24 835–850. 10.1111/mec.13067 25581031

[B136] Yu A. Lepère G. Jay F. Wang J. Bapaume L. Wang Y. (2013). Dynamics and biological relevance of DNA demethylation in *Arabidopsis* antibacterial defense. *Proc. Natl. Acad. Sci. U.S.A.* 110 2389–2394. 10.1073/pnas.1211757110 23335630 PMC3568381

[B137] Zas R. Cendán C. Sampedro L. (2013). Mediation of seed provisioning in the transmission of environmental maternal effects in Maritime pine (*Pinus pinaster* Aiton). *Heredity* 111 248–255. 10.1038/hdy.2013.44 23652562 PMC3746824

[B138] Zhang H. Zhu J.-K. (2011). RNA-directed DNA methylation. *Curr. Opin. Plant Biol* 14 142–147. 10.1016/j.pbi.2011.02.003 21420348 PMC3096526

[B139] Zhang H. Zhu J.-K. (2012). Active DNA demethylation in plants and animals. *Cold Spring Harb. Symp. Quant. Biol.* 77 161–173. 10.1101/sqb.2012.77.014936 23197304 PMC3657592

[B140] Zhang R. Gallagher R. Shea K. (2012). Maternal warming affects early life stages of an invasive thistle. *Plant Biol.* 14 783–788. 10.1111/j.1438-8677.2011.00561.x 22404764

[B141] Zhang Y. Y. Fischer M. Colot V. Bossdorf O. (2013). Epigenetic variation creates potential for evolution of plant phenotypic plasticity. *New Phytol.* 197 314–322. 10.1111/nph.12010 23121242

[B142] Zheng X. Chen L. Li M. Lou Q. Xia H. Wang P. (2013). Transgenerational variations in DNA methylation induced by drought stress in two rice varieties with distinguished difference to drought resistance. *PLoS One* 8:e80253. 10.1371/journal.pone.0080253 24244664 PMC3823650

